# Development and Evaluation of a New Frailty Index for Older Surgical Patients With Cancer

**DOI:** 10.1001/jamanetworkopen.2019.3545

**Published:** 2019-05-10

**Authors:** Armin Shahrokni, Amy Tin, Koshy Alexander, Saman Sarraf, Anoushka Afonso, Olga Filippova, Jennifer Harris, Robert J. Downey, Andrew J. Vickers, Beatriz Korc-Grodzicki

**Affiliations:** 1 Geriatrics Service, Department of Medicine, Memorial Sloan Kettering Cancer Center, New York, New York; 2 Department of Epidemiology and Biostatistics, Memorial Sloan Kettering Cancer Center, New York, New York; 3 Department of Anesthesiology and Critical Care Medicine, Memorial Sloan Kettering Cancer Center, New York, New York; 4 Gynecology Service, Department of Surgery, Memorial Sloan Kettering Cancer Center, New York, New York; 5 Hepatopancreatobiliary Service, Department of Surgery, Memorial Sloan Kettering Cancer Center, New York, New York; 6 Thoracic Service, Department of Surgery, Memorial Sloan Kettering Cancer Center, New York, New York

## Abstract

**Importance:**

Frailty based on the modified Frailty Index is associated with poor postoperative outcomes. However, the index requires high levels of personnel time and effort and often has missing data.

**Objective:**

To evaluate the association of the Memorial Sloan Kettering–Frailty Index (MSK-FI) with established geriatric assessment (GA) and surgical outcomes.

**Design, Setting, and Participants:**

This cohort study included prospectively evaluated patients with cancer 75 years and older who were referred to MSK Geriatrics Service clinics for preoperative evaluation before undergoing surgery requiring hospitalization between February 2015 and September 2017. Patients were comanaged by the Geriatrics Service and Surgery Service in the postoperative period.

**Exposures:**

Impairments identified by GA and comorbid conditions retrieved from submitted *International Classification of Diseases, Ninth Revision *(*ICD-9*) and *ICD-10* codes within the first 48 hours of hospitalization.

**Main Outcomes and Measures:**

The association of MSK-FI score (which included *ICD-9* and *ICD-10* codes) with GA impairments (based on clinical interview and examination as well as patient reports) was examined. The associations of MSK-FI score with short-term surgical outcomes (ie, frequency of complications, length of stay, 30-day surgical complications, 30-day intensive care unit admissions, and 30-day readmissions) and 1-year survival, estimated by Kaplan-Meier methods, were determined.

**Results:**

In total, 1137 patients (median [interquartile range] age, 80 [77-84] years; 583 [51.2%] women) were included in the study. A higher MSK-FI score was associated with the number of GA impairments (ρ = 0.52; bootstrapped 95% CI, 0.47-0.56). Each 1-point increase in MSK-FI score was associated with longer length of stay (0.58 d; 95% CI, 0.22-0.95; *P* = .002) and higher odds of intensive care unit admission (odds ratio, 1.28; 95% CI, 1.04-1.58; *P* = .02). Median (interquartile range) follow-up among survivors was 12.1 (5.6-19.1) months. The MSK-FI score was associated with overall mortality; 12-month risk of death was 5% for a score of 0 and approximately 20% for scores of 4 and higher (nonlinear association, *P* = .005).

**Conclusions and Relevance:**

In this study, the MSK-FI was associated with the previously validated GA and postoperative outcomes in older patients with cancer and may be a feasible tool for perioperative assessment of older surgical patients with cancer. Future studies should assess the association of MSK-FI score with postoperative care and outcomes of older, frail patients with cancer.

## Key Points

**Question:**

Can a more clinically feasible version of the modified Frailty Index for older patients with cancer be developed?

**Findings:**

In this cohort study of 1137 older patients with cancer, the Memorial Sloan Kettering–Frailty Index (MSK-FI) was associated with aging-related impairments. A higher score on the MSK-FI was also associated with a longer length of stay, higher odds of intensive care unit admission, and lower overall survival.

**Meaning:**

The MSK-FI may be a feasible tool to perioperatively assess frailty in older patients with cancer.

## Teaser

This cohort study evaluates the association of the Memorial Sloan Kettering–Frailty Index with established geriatric assessment and surgical outcomes for older surgical patients with cancer.

## Introduction

Although surgery remains the primary treatment modality for most localized solid tumors, it can do more harm than good in some older patients. It is clear that chronologic age alone should not be used to make decisions regarding the ability of a patient to recover from surgery; surgeons treating older patients with cancer should take into account factors other than age that predict the likelihood of poor postoperative outcomes.^1^ Frailty is a clinical syndrome defined by decreased physiological capabilities and reserve to maintain homeostasis after a stressor,^2^ and multiple studies have shown an important association of preoperative frailty with adverse surgical outcomes.^3,4,5^

The geriatric assessment (GA) is considered the criterion standard for assessing frailty, and it has been shown to accurately identify patients at risk of postoperative adverse events.^1,6^ However, conducting GA is time-consuming and may not be feasible in an oncology practice with limited resources. Additionally, most surgeons have limited training in implementing and interpreting GA results^7^ and may need to rely on health care professionals specializing in geriatric health care.^1^

In 2007, investigators developed a 70-item Frailty Index based on the Canadian Study of Health and Aging.^8^ Subsequently, the modified Frailty Index (mFI)^9^ was developed using 11 of the 70 items in the Canadian Study of Health and Aging Frailty Index. Those 11 items were initially available in the National Surgical Quality Improvement Program (NSQIP) database, to which 601 US hospitals report. Of the 11 items included in the mFI, 10 are related to comorbid conditions, and 1 is related to the patient’s functional status (basic and instrumental activities of daily living). Patients who have an mFI score of 3 or more (1 point for dependent functional status and 1 point for each comorbidity) are considered frail.^9^ Frailty (according to the mFI) has been shown to be a better predictor of mortality than age or American Society of Anesthesiologists (ASA) score,^10^ and higher scores are associated with postoperative complications.^9,10,11^

However, the mFI has significant limitations, primarily in feasibility. The index depends on NSQIP data collection. Unfortunately, in recent years, some NSQIP variables have been removed from the database. Furthermore, while the 11 variables used for the mFI were originally mandatory for reporting to NSQIP, none has been mandatory since 2012. As a result, the number of missing items has increased significantly. Gani et al^12^ evaluated the increase of missing data for 18 159 patients who underwent a pancreaticoduodenectomy between 2005 and 2013. The authors found that missing data for 5 of the 11 mFI variables increased over time, with 55% of data missing in 2011 and 100% missing in 2013.^12^ They found similar results for other procedures; 44% of data for esophagectomy and 67% for total hip replacement were missing in 2011, and in 2013, 100% of data were missing for both procedures.^12^

A reliable source of data that includes the variables from the original mFI and that is not likely to be discontinued in the future is needed. In this article, we aim to develop a new frailty index (the Memorial Sloan Kettering–Frailty Index [MSK-FI]) that is associated with the criterion standard of frailty, the GA, and with multiple postoperative parameters as well as be easy to use and place minimal additional burden on personnel.

## Methods

### Patients

At the MSK Cancer Center, patients 75 years and older in need of preoperative evaluation are referred to the Geriatrics Service clinic. As part of this preoperative evaluation, patients complete an electronic version of the GA, the electronic Rapid Fitness Assessment (eRFA), which incorporates questions related to functional ability, emotional well-being (distress and depression), cognition, presence of social support, level of social activity, nutritional status, medications, and sensory deficits.^13^ Data from the eRFA are prospectively collated in an electronic database. All patients evaluated by the Geriatrics Service are comanaged by the Geriatrics Service and the patient’s primary surgical service during the inpatient postoperative period. Following hospital discharge, patients are followed up with by the primary surgical service. From a prospectively maintained Geriatrics Service database, we identified 1160 patients who completed the GA during or before their geriatric clinic visit, underwent a surgical procedure within 60 days of completing their GA, and had a postoperative length of stay (LOS) of at least 1 day between February 2015 and September 2017. We excluded 8 patients with missing data on basic or instrumental activities of daily living (used to determine functional status dependency) and 15 patients with missing values for other covariates that we planned to include in our model (9 patients had missing ASA scores; 6 patients had missing albumin level measurements from within 60 days before surgery). Our final cohort consisted of 1137 patients.

The MSK institutional review board approved a waiver of authorization to allow performance of this study; patient consent was not required. This article adheres to the Strengthening the Reporting of Observational Studies in Epidemiology (STROBE) reporting guideline for cohort studies.

### Development of the MSK-FI

Using the eRFA data collected on older patients undergoing preoperative evaluation, we developed the MSK-FI on the basis of the parameters of the mFI. Both the MSK-FI and the mFI include preoperative functional status and comorbid conditions.

Similar to the mFI, the MSK-FI functional status assessment is based on 4 basic patient-reported activities of daily living (ie, bathing, dressing, grooming, and walking outside the home) and 1 instrumental activity of daily living (ie, preparing meals). Limitation in any of these activities is considered “impaired functional activity” and is given a value of 1; patients who are independent (ie, able to complete all basic and instrumental activities of daily living) receive a value of 0.

The 10 comorbidities used in the MSK-FI (which are identical to the administrative staff–abstracted comorbidities included in the mFI) are (1) chronic obstructive pulmonary disease or pneumonia within 30 days before surgery, (2) diabetes, (3) congestive heart failure, (4) myocardial infarction, (5) coronary artery disease, (6) hypertension, (7) peripheral vascular disease, (8) impaired sensorium (which incorporates Alzheimer disease, delirium, dementia, Lewy body disease, mild cognitive impairment, and memory loss), (9) cerebrovascular accident, and (10) transient ischemic attack. Unlike the mFI, the comorbid conditions for the MSK-FI are retrieved from *International Classification of Diseases, Ninth Revision *(*ICD-9*) and *ICD-10* codes that are submitted until 2 days after surgery. The score for comorbid conditions ranges from 0 to 10, with 1 point assigned for the presence of each condition. The MSK-FI final score is based on the combined patient-reported functional assessment and the presence of the 10 comorbid conditions from *ICD-9 *and *ICD-10* codes, with the total score ranging from 0 to 11.

### Association of MSK-FI With GA

**Figure 1.**
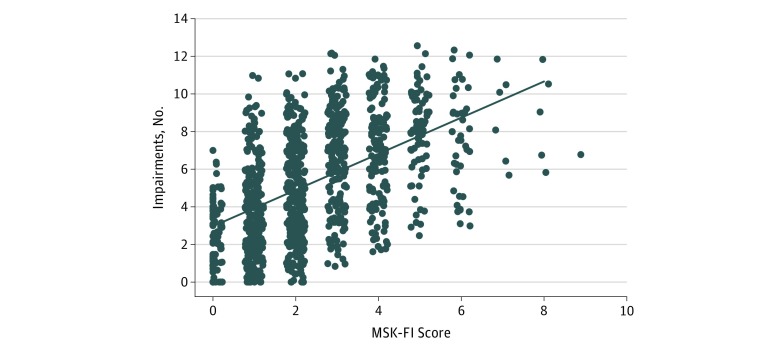
Scatterplot of Total Impairments by Memorial Sloan Kettering–Frailty Index (MSK-FI) Scores Points have been shifted randomly to minimize overlap. The linear prediction line is included for visualization purposes. Spearman correlation is 0.52 (bootstrapped 95% CI, 0.47-0.56).

Figure 1 visualizes the association of the number of impairments identified on the basis of 13 components of the GA (score range, 0-13; eTable in the Supplement), in accordance with the Rockwood theory of cumulative deficit,^14^ with MSK-FI score. Spearman correlation and 95% CIs are presented.

### Association of MSK-FI With Surgical Outcomes

Surgical outcomes included postoperative LOS, frequency of major complications, readmission rate, intensive care unit (ICU) admissions (all within 30 days after surgery), and overall survival, particularly 1-year survival as estimated by Kaplan-Meier methods. These were collected from electronic medical records. Major complications were defined on the basis of the Clavien-Dindo classification.^15^ At MSK, complications and their severity are collected every 30 days by attending surgeons, house staff, and research staff. A 2015 study^16^ showed the high accuracy of such mechanisms in reporting major complications. Major complications are defined as grade 3 and higher complications that require radiologic, endoscopic, or operative intervention (grade 3), chronic disability or organ resection (grade 4), or death (grade 5).

### Statistical Analysis

Univariable regression was used to determine the association of MSK-FI score with each of the outcomes, and multivariable regression (adjusted for age at surgery, operating room duration, ASA score [2 vs 3 vs 4],^17,18^ preoperative albumin level^19,20^ [included in the model as a nonlinear term], and the presence of polycomorbid conditions) was used to determine whether the association remained significant after adjustment for standard factors associated with outcomes. *P* < .05 was considered statistically significant, and tests were 2-tailed. Linear regression was used for LOS; logistic regression was used for readmissions and ICU admissions within 30 days of surgery (separately); and Cox regression was used for overall survival. For each outcome, we tested the MSK-FI score for nonlinearity using restricted cubic splines with knots at the tertiles. We found no evidence of a nonlinear association of MSK-FI score with LOS, readmission, or ICU admission; therefore, the MSK-FI score was included in the model as a linear variable. However, for the outcome of overall survival, we did observe evidence of a nonlinear association of MSK-FI score with overall survival; therefore, the MSK-FI score was included in the model as a nonlinear term using restricted cubic splines with knots at the tertiles. For overall survival, we depicted the 12-month survival probability across the range of MSK-FI scores. Patients were censored at the date they were last known to be alive.

Because the association of MSK-FI score with outcomes could vary with the type of surgery, we examined whether stratification by surgical category (Table 1) affected our findings. For each surgical category, we used the same multivariable regression for each outcome of interest and extracted the effect size. As we previously observed evidence of a nonlinear association of the MSK-FI score with overall survival—and to enter only 1 term, rather than the cubic splines, to allow extraction of the effect size—the MSK-FI score was entered as a binary term (using the cutoff value for frailty from the mFI of 3) in the multivariable regression models for overall survival. The effect sizes for each surgical category were then combined using fixed effects, with the weights calculated using inverse-variance weighting. Using these estimates, we tested for heterogeneity, where the null hypothesis was that the association of the corresponding outcome with MSK-FI score does not differ by surgical category. Finally, we performed sensitivity analyses on the association of MSK-FI score with 1-year survival, excluding patients with stage IV cancer first, then stage III and IV cancer, as their natural courses of disease differ from those with more localized cancer. All statistical analyses were conducted using Stata version 15.0 (StataCorp).

**Table 1.** Demographic Characteristics of 1137 Patients

Characteristic	No. (%)
Age at surgery, median (IQR), y	80 (77-84)
Women	583 (51.3)
Race	
White	961 (84.5)
Black	49 (4.3)
Asian	63 (5.5)
Other^a^	15 (1.3)
Unknown	49 (4.3)
MSK-FI score	
0	87 (7.7)
1	269 (23.7)
2	314 (27.6)
3	211 (18.6)
4	136 (12.0)
≥5	120 (10.6)
Preoperative albumin level, median (IQR), g/dL	4.0 (3.8-4.3)
Operating room duration, median (IQR), min	163 (103-250)
ASA physical status classification	
2	73 (6.4)
3	977 (85.9)
4	87 (7.7)
Surgical category^b^	
Colorectal	460 (40.5)
Gastric and mixed tumor	69 (6.1)
Gynecology	280 (24.6)
Head and neck	299 (26.3)
Interventional radiology	158 (13.9)
Urology	202 (17.8)
Neurosurgery	72 (6.3)
Plastic	99 (8.7)
Hepatobiliary and pancreatic	190 (16.7)
Thoracic	163 (14.3)
Other	178 (15.7)

Abbreviations: ASA, American Society of Anesthesiologists; IQR, interquartile range; MSK-FI, Memorial Sloan Kettering–Frailty Index.

SI conversion factor: To convert albumin to grams per liter, multiply by 10.

^a^ Other category included Native American, Native Hawaiian, Alaskan Native, and Pacific Islander.

^b^ Percentages do not sum to 100, as patients underwent multiple procedures under different categories.

## Results

Among our cohort of 1137 patients, 583 (51.2%) were women, and the median (interquartile range [IQR]) age at surgery was 80 (77-84) years. Additional patient demographic characteristics are presented in Table 1. Figure 1 depicts the association of MSK-FI score with the number of GA impairments (ρ = 0.52; bootstrapped 95% CI, 0.47-0.56). The components of the GA (and 1 component related to comorbidities) and the MSK-FI score, where higher MSK-FI score indicates greater frailty, are shown in eFigure 1, eFigure 2, and eFigure 3 in the Supplement. The median MSK-FI score was higher among patients who were dependent for the basic and instrumental activities of daily living, experienced a fall in the past year, had poor Karnofsky performance status scores, had slower gait speed, had limited social activity, were taking 5 or more medications, or had 4 or more comorbid conditions compared with patients without impairments in those domains.

Among our cohort, the median (IQR) LOS was 5 (3-8) days, and 96 patients (8.4%; 95% CI, 6.9%-10.2%) were readmitted, 54 patients (4.7%; 95% CI, 3.6%-6.2%) were admitted to the ICU, and 65 patients (5.7%; 95% CI, 4.4%-7.2%) had major complications within 30 days of surgery. Table 2 depicts the association of MSK-FI score with each outcome. On multivariable analysis, a 1-point increase in MSK-FI score was associated with longer LOS (0.58 d; 95% CI, 0.22-0.95; *P* = .002). Additionally, MSK-FI score was associated with higher odds of ICU admission (odds ratio, 1.28; 95% CI, 1.04-1.58; *P* = .02). For instance, with all covariates set to the mean, a patient with an MSK-FI score of 2 would have a 3.0% rate of ICU admission and a patient with an MSK-FI score of 4 would have a 4.9% rate of ICU admission.

**Table 2.** Association of Memorial Sloan Kettering–Frailty Index Score With Surgical Outcomes^a^

Variable	Estimate (95% CI)^b^	*P* Value	*P* Value for Heterogeneity^c^
Length of stay	0.58 (0.22-0.95)	.002	.37
Readmission within 30 d of surgery	0.98 (0.82-1.17)	.84	.26
ICU admission within 30 d of surgery	1.28 (1.04-1.58)	.02	.98
Major complications^d^	1.04 (0.84-1.29)	.70	.77^d^

Abbreviation: ICU, intensive care unit.

^a^ Adjusted for age at surgery, operating room duration, American Society of Anesthesiologists score, preoperative albumin level (included in the model as a nonlinear term), and the presence of polycomorbid conditions.

^b^ Estimate corresponds to β (linear regression model for the outcome of length of stay) and odds ratio (logistic regression model for the outcomes of readmission and ICU admission) for a 1-point change on the frailty index. For Cox regression model for overall survival, *P* = .005.

^c^ The heterogeneity *P* value tests the null hypothesis that the association of the outcome with Memorial Sloan Kettering–Frailty Index score does not differ on the basis of type of surgical procedure. Statistical significance set at *P* < .05.

^d^ This analysis excluded 72 patients undergoing neurosurgery procedures, as only 1 patient had a major complication.

**Figure 2.**
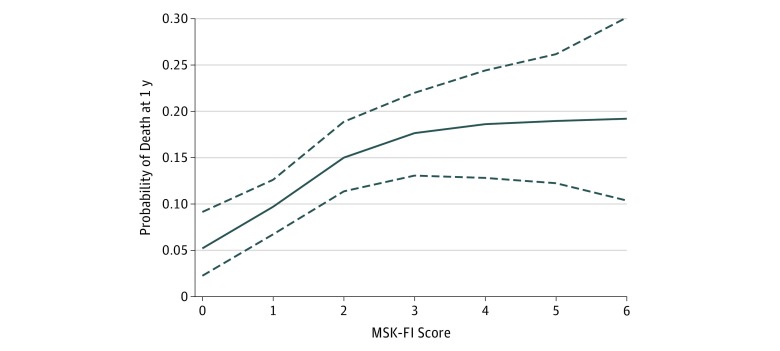
Estimated Probability of Death at 1 Year Of 1137 participants, 12 (6 with Memorial Sloan Kettering–Frailty Index [MSK-FI] score of 7; 5 with MSK-FI score of 8; and 1 with MSK-FI score of 9) are not shown. Covariates are set at the mean. Dotted lines represent bootstrapped 95% CIs.

Overall, there were 189 deaths in our cohort, of which 129 were within 1 year of surgery. The median (IQR) postoperative follow-up for survivors was 12.1 (5.6-19.1) months. Figure 2 depicts the estimated probability of death at 12 months laid over the distribution of MSK-FI scores, where we see that MSK-FI score was associated with worse overall survival. In particular, there was an increase in the probability of death as MSK-FI score increased from 0 to 4. The 12-month risk of death was 5% for an MSK-FI score of 0 compared with nearly 20% for an MSK-FI score of 4 or higher (nonlinear association, *P* = .005). Results of sensitivity analyses showed that the association of MSK-FI with 1-year mortality persisted after patients with stage IV cancer or stage III/IV cancer (eFigure 4 and eFigure 5 in the Supplement, respectively) were excluded.

For our cohort of patients, MSK-FI score was not significantly associated with the rates of major complications (HR, 1.04; 95% CI, 0.84-1.29; *P* = .70) or readmissions (HR, 0.98, 95% CI, 0.82-1.17; *P* = .84). Results were similar on univariable analysis, and there was no evidence of heterogeneity for any outcome when stratified by surgical category (Table 2).

## Discussion

Using prospectively collected data on a large cohort of older patients with cancer who presented to the MSK Geriatrics Service for preoperative evaluation for surgery that required hospitalization, we developed a new frailty index, the MSK-FI. This index is specifically designed to compensate for missing comorbidity-associated variables in the NSQIP database, and it does this by harvesting data relevant to the missing fields from the *ICD*-9 and *ICD*-*10* codes used for hospital billing. Hospitals and emergency departments rely on correct classification of *ICD*-9 and *ICD*-*10* codes for proper reimbursement, clinical documentation, case-mix acuity indices, medical necessity for procedures, services and admissions, and reporting of disease to public health departments.^21^

To determine the association of the MSK-FI with the GA, we assessed the association of the MSK-FI score of patients with the number of impairments identified on the basis of the GA. We found that there was a moderate association of MSK-FI score with number of impairments (ρ = 0.52; 95% CI, 0.47-0.56). Moreover, we observed higher MSK-FI scores among patients who were dependent for the basic and instrumental activities of daily living, experienced a fall in the past year, had poor Karnofsky performance status scores, had slower gait speed, had limited social activity, were taking 5 or more medications, or had 4 or more comorbid conditions.

We found that frail patients (as defined by higher MSK-FI score) were likely to experience longer LOSs, increased frequency of postoperative ICU admissions, and decreased overall survival. These findings are consistent with previous reports.^22,23^ Future studies should assess the potential role of the MSK-FI in postoperative care processes and treatment decision making, such as determining candidates for and timing of adjuvant treatment.

We did not observe an association of frailty with rate of complications after surgery or need for 30-day readmissions, which is in contrast with other studies that found an association of frailty with these outcomes.^6,24^ A reason for the lack of association in our study could be related to the compensatory care offered to patients who are frail.^25^ Our cohort consisted of older patients who presented to Geriatrics Service for preoperative evaluation and were subsequently comanaged by the Geriatrics Service and Surgery Service during the postoperative period. Postoperative comanagement of older adults in nononcologic surgical settings has been shown to be effective in reducing adverse surgical events. For example, a systematic review of 18 studies on comanagement of older patients between geriatricians and orthopedists^26^ found that comanagement reduced in-hospital mortality, long-term mortality, and hospital LOS. A study evaluating 319 patients 65 years and older who were hospitalized for hip fracture^27^ found that geriatric comanagement reduced LOS by 2 days, decreased in-hospital mortality (0.6% vs 5.8%), and reduced the major complication rate (45% vs 62%).

The MSK-FI can be used to determine frailty in the perioperative period and could be used when providing enhanced postoperative care for frail patients, with the aim of reducing hospital LOS and postoperative ICU admissions. It can be extremely useful at the administrative level to predict the acuity of postoperative medical needs of older patients with cancer, and, hence, to proactively plan for adequate staffing and appropriate support for those older frail patients with cancer at a higher risk of poor surgical outcomes. Interventions such as transitional care management and closer outpatient follow-up by the patient’s primary care clinician or geriatrician, in addition to surgical services, could potentially improve the overall survival of these patients. The MSK-FI could be easily implemented in various institutions and surgical services. The only added burden would be to assess patients’ ability to perform basic and instrumental activities of daily living, since all additional data that are needed for the MSK-FI can be retrieved using *ICD*-9 and *ICD*-*10* codes. Wider implementation of the MSK-FI will allow for the comparison of surgical outcomes in different institutions based on the frailty status of patients.

### Limitations and Strengths

This study has several limitations. This is a single-institution study conducted at a comprehensive cancer center that evaluated a cohort of older patients with cancer who were referred by the Surgery Service to the Geriatrics Service for preoperative evaluation. The association of frailty with outcomes could have been underestimated in our study, given the surgical specialization, patient volume, use of geriatric care, and other resources that may not be present at other institutions. Future studies should assess the validity and utility of the MSK-FI in institutions with limited resources, especially among institutions in which postoperative geriatric comanagement of older patients with cancer is not routine. Among our cohort, only a relatively small number of patients had very high MSK-FI scores. Moreover, our study included only patients 75 years and older. Future studies should assess the utility of the MSK-FI in assessing frailty among patients younger than 75 years, as well as among patients with a higher degree of frailty. Our study could have been strengthened by adjusting the association of the MSK-FI score with surgical outcomes by preoperative blood work results, intraoperative variables (such as hemodynamic status and use of pressors), and status of postoperative health care processes (such as physical or occupational therapy). Also, in our multivariable model, we did not include the receipt of adjuvant or neoadjuvant treatment, which could affect postoperative outcomes of older patients with cancer. In future studies, we will assess the postoperative outcomes of more homogeneous subcohorts on the basis of the MSK-FI and the receipt of adjuvant and neoadjuvant treatments. We also emphasize that, at this point, the MSK-FI is not a preoperative risk stratification tool because the comorbid conditions are retrieved from claims submitted within the first 48 hours of hospital admission after surgery. We strongly recommend that, when feasible, the preoperative assessment of older patients with cancer include GA to ensure full assessment of frailty in these patients. However, the value of the MSK-FI is in designing postoperative interventions aimed at improving postoperative outcomes of frail older patients with cancer.

Nonetheless, our study also has several strengths. It includes one of the largest cohorts of patients with cancer 75 years or older assessed for surgical outcomes. Our analysis included a robust amount of data on patients’ overall preoperative fitness as well as their GA. Geriatrics preoperative evaluation and GA were performed by geriatricians as routine perioperative management; as a result, there is minimal possibility of health selection bias, which tends to occur in observational studies.^28^ This also eliminated the need for random selection of patients, which is a characteristic of analyses of NSQIP data.

## Conclusions

In this study, the MSK-FI was shown to be associated with previously validated GA outcomes and postoperative outcomes in older patients with cancer. This tool may be used to perioperatively assess frailty among older patients with cancer. Future studies should seek to externally validate this tool, particularly with respect to its use for postoperative health care and outcomes.

## Notes

Supplement. **eTable.** Twelve Components of the Electronic Rapid Fitness Assessment and the Definition of Polycomorbid Conditions **eFigure 1.** Boxplots of Functional Components of the Geriatric Assessment, Based on MSK-FI **eFigure 2.** Boxplots of Emotional and Social Components of the Geriatric Assessment, Based on MSK-FI **eFigure 3.** Boxplots of Overall Health Components of the Geriatric Assessment, Based on MSK-FI **eFigure 4.** After Excluding Patients With Stage IV Cancer, Estimated Probability of Death at 12 Months (With Covariates Set at the Mean and Bootstrapped 95% CI) **eFigure 5.** After Excluding Patients With Stage III or IV Cancer, Estimated Probability of Death at 12 Months (With Covariates Set at the Mean and Bootstrapped 95% CI) **eReferences**
