# Supplementary material for: Development and Evaluation of a New Frailty Index for Older Surgical Patients With Cancer
Source: JAMA Netw Open. 2019 May 10;2(5):e193545. doi: 10.1001/jamanetworkopen.2019.3545 (PMC6512296; doi:10.1001/jamanetworkopen.2019.3545)
Supplement: Supplement. — eTable. Twelve Components of the Electronic Rapid Fitness Assessment and the Definition of Polycomorbid Conditions eFigure 1. Boxplots of Functional Components of the Geriatric Assessment, Based on MSK-FI eFigure 2. Boxplots of Emotional and Social Components of the Geriatric Assessment, Based on MSK-FI eFigure 3. Boxplots of Overall Health Components of the Geriatric Assessment, Based on MSK-FI eFigure 4. After Excluding Patients With Stage IV Cancer, Estimated Probability of Death at 12 Months (With Covariates Set at the Mean and Bootstrapped 95% CI) eFigure 5. After Excluding Patients With Stage III or IV Cancer, Estimated Probability of Death at 12 Months (With Covariates Set at the Mean and Bootstrapped 95% CI) eReferences [file jamanetwopen-2-e193545-s001.pdf]

Supplementary Online Content

Shahrokni A, Tin A, Alexander K, et al. Development and evaluation of a new frailty index for older surgical patients with cancer. *JAMA Netw Open.* 2019;2(5):e193545. doi:10.1001/jamanetworkopen.2019.3545

eTable. Twelve Components of the Electronic Rapid Fitness Assessment and the Definition of Polycomorbid Conditions
eFigure 1. Boxplots of Functional Components of the Geriatric Assessment, Based on MSK-FI
eFigure 2. Boxplots of Emotional and Social Components of the Geriatric Assessment, Based on MSK-FI
eFigure 3. Boxplots of Overall Health Components of the Geriatric Assessment, Based on MSK-FI
eFigure 4. After Excluding Patients With Stage IV Cancer, Estimated Probability of Death at 12 Months (With Covariates Set at the Mean and Bootstrapped 95% CI)
eFigure 5. After Excluding Patients With Stage III or IV Cancer, Estimated Probability of Death at 12 Months (With Covariates Set at the Mean and Bootstrapped 95% CI)
eReferences

This supplementary material has been provided by the authors to give readers additional information about their work.

eTable. Twelve Components of the Electronic Rapid Fitness Assessment and the Definition of Polycomorbid Conditions

Domain	Instrument	Description	Score range	Cutoff score
**Functional Domain**	**bADL**[1]	Assesses patients' level of independence in performing seven activities: bathing, dressing, grooming, feeding, bladder and bowel control, and walking inside and outside of the house. Patients were able to state whether, in any of these activities, they were limited a lot, limited a little, or not limited at all.	0-14	<14
	**iADL**[2]	Assesses patients' level of independence in eight activities: telephone use, doing laundry, shopping, preparing meals, doing housework, handling own medications, handling money and finances, and transportation. Patients were able to state whether they were unable to perform any of these activities, needed some help, or did not need any help.	0-16	<16
	**Patient-rated KPS**[3]	NA	30-100	≤80
	**Fall in the past year**	NA	None, One time, More than one time	Patients who experienced at least one fall in the past year
	**Timed Up and Go test** [4]	Patients are asked by the Geriatrics nurses to stand from the chair, walk 10 feet, turn, and return to the chair.	<10 seconds, 10-19 seconds, ≥20 seconds	≥10 seconds
**Social Support/Activity**	**Social Support Survey**[5]	Social Support: Measured by Medical Outcome Study-Social Support Survey-4 item.	4-20	≤16
	**Social activity limitation**[6]	Limited Social Activity: Measured by Medical Outcome Study-Social Activity Survey.	3-15	≥8
**Cognition**	**Mini-Cog**[7]	Patients are told 3 words to memorize. Patients are then asked to draw a clock with hands showing 10 past 11. Patients are asked to recall the 3 words. Normal/abnormal clock drawing is scored as 2/0 points, and 1 point is given for each correctly recalled word (total of 3 points).	0-5	≤2
**Emotional Well-being**	**Distress**[8]	Measured by Distress Thermometer. Patients are asked to rate their distress level from 1 to 10.	1-10	≥4
	**Depression**[9]	Measured by Geriatric Depression Scale-4 item questionnaire	0-4	≥1

© 2019 Shahrokni A et al. *JAMA Network Open.*

**Polypharmacy**		Patients report the number of medications they take.	1-4 medications,<br>5-9 medications,<br>≥10 medications	≥5 medications
**Nutritional Status**		Weight change in the past 6 months	1, no change or weight gain<br>2, lost <5 lbs<br>3, lost 5-10 lbs<br>4, lost 10-20 lbs<br>5, lost ≥20 lbs	≥10 pounds
**Polycomorbid Conditions**		Comorbid conditions: Retrieved from electronic medical record using ICD9 and 10 codes. Presence of 4 or more comorbid conditions is considered abnormal.<br><br>The 13 comorbid conditions of interest were:<br>1. Coronary artery disease, or atrial fibrillation, or atrial flutter, or myocardial infarction, or cardiomyopathy, or heart failure<br>2. Dementia, or Alzheimer's, or Lewy body disease, or memory loss, or MCI, or delirium<br>3. Arthritis or degenerative joint disease<br>4. Chronic obstructive pulmonary disease/asthma or pneumonia<br>5. Cerebrovascular accident or transient ischemic attack<br>6. Diabetes<br>7. Hypertension<br>8. Kidney failure<br>9. Lipid disorders<br>10. Liver diseases<br>11. Peptic ulcer disorder<br>12. Peripheral vascular disease<br>13. Thyroid diseases	0-13	≥4

bADL, basic activities of daily living; iADL, instrumental activities of daily living; KPS, Karnofsky Performance Status; MCI, mild cognitive imparment; NA, not applicable.

© 2019 Shahrokni A et al. *JAMA Network Open.*

eFigure 1. Boxplots of Functional Components of the Geriatric Assessment, Based on MSK-FI

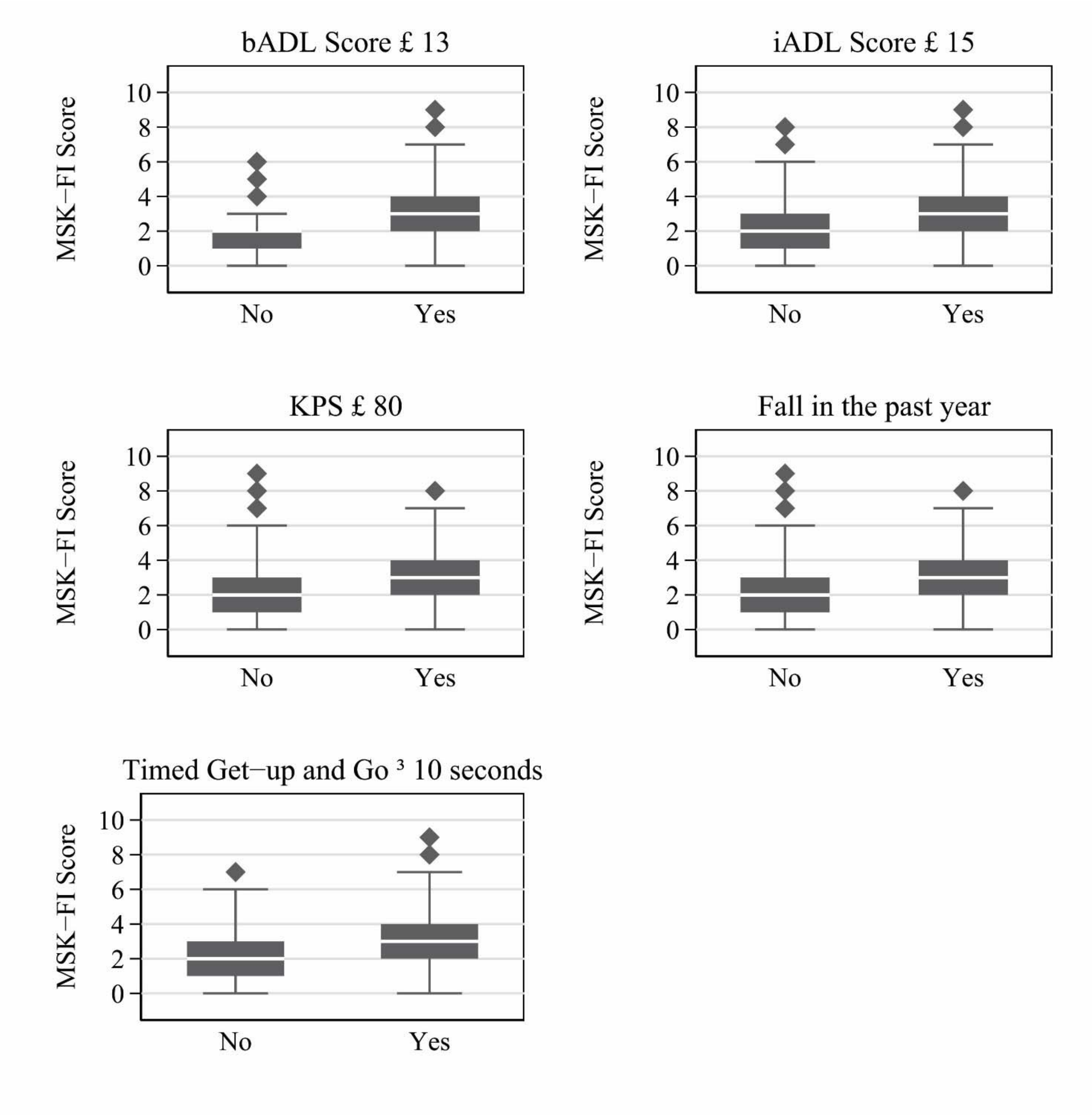

© 2019 Shahrokni A et al. *JAMA Network Open.*

eFigure 2. Boxplots of Emotional and Social Components of the Geriatric Assessment, Based on MSK-FI

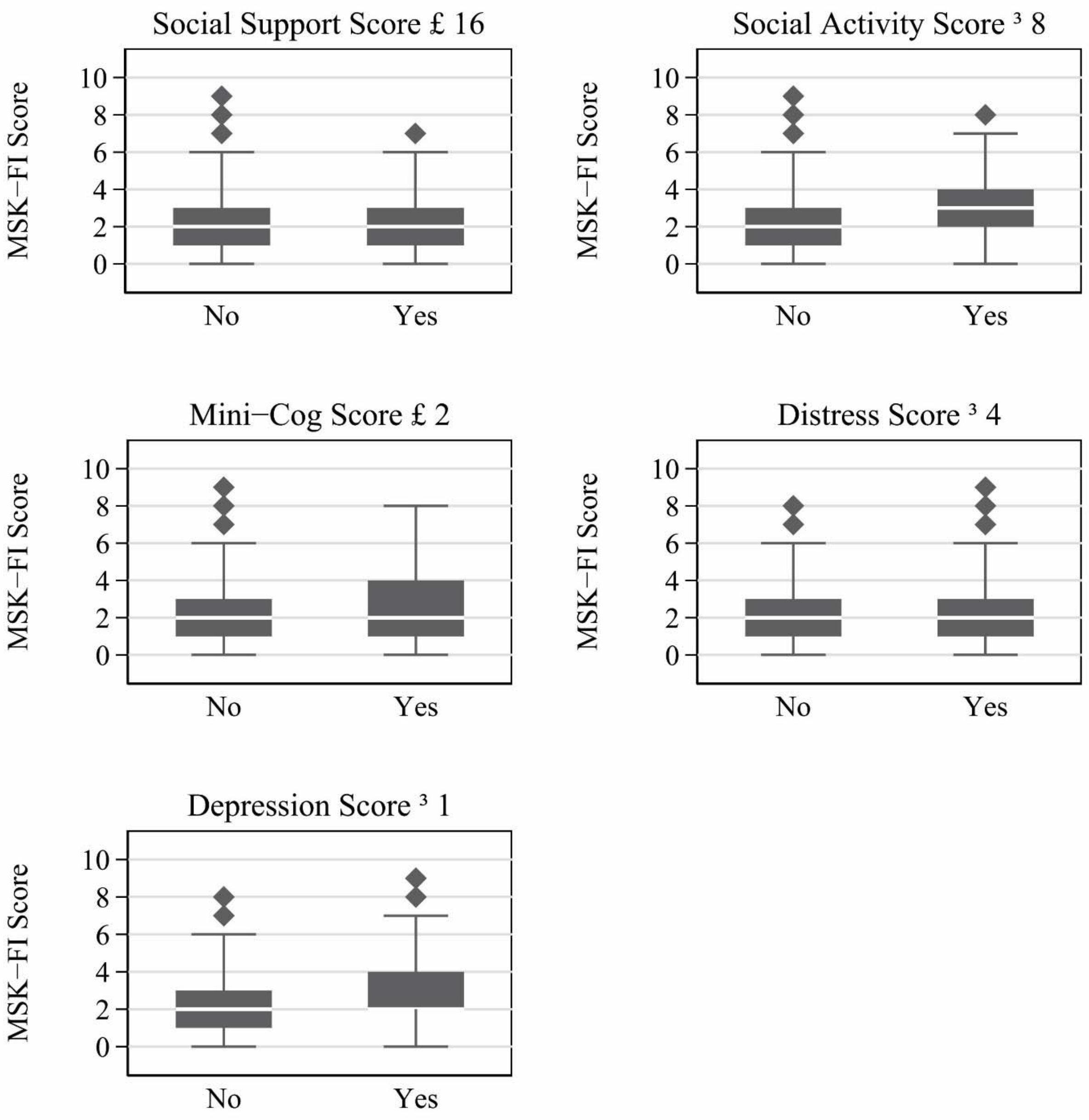

© 2019 Shahrokni A et al. *JAMA Network Open.*

eFigure 3. Boxplots of Overall Health Components of the Geriatric Assessment, Based on MSK-FI

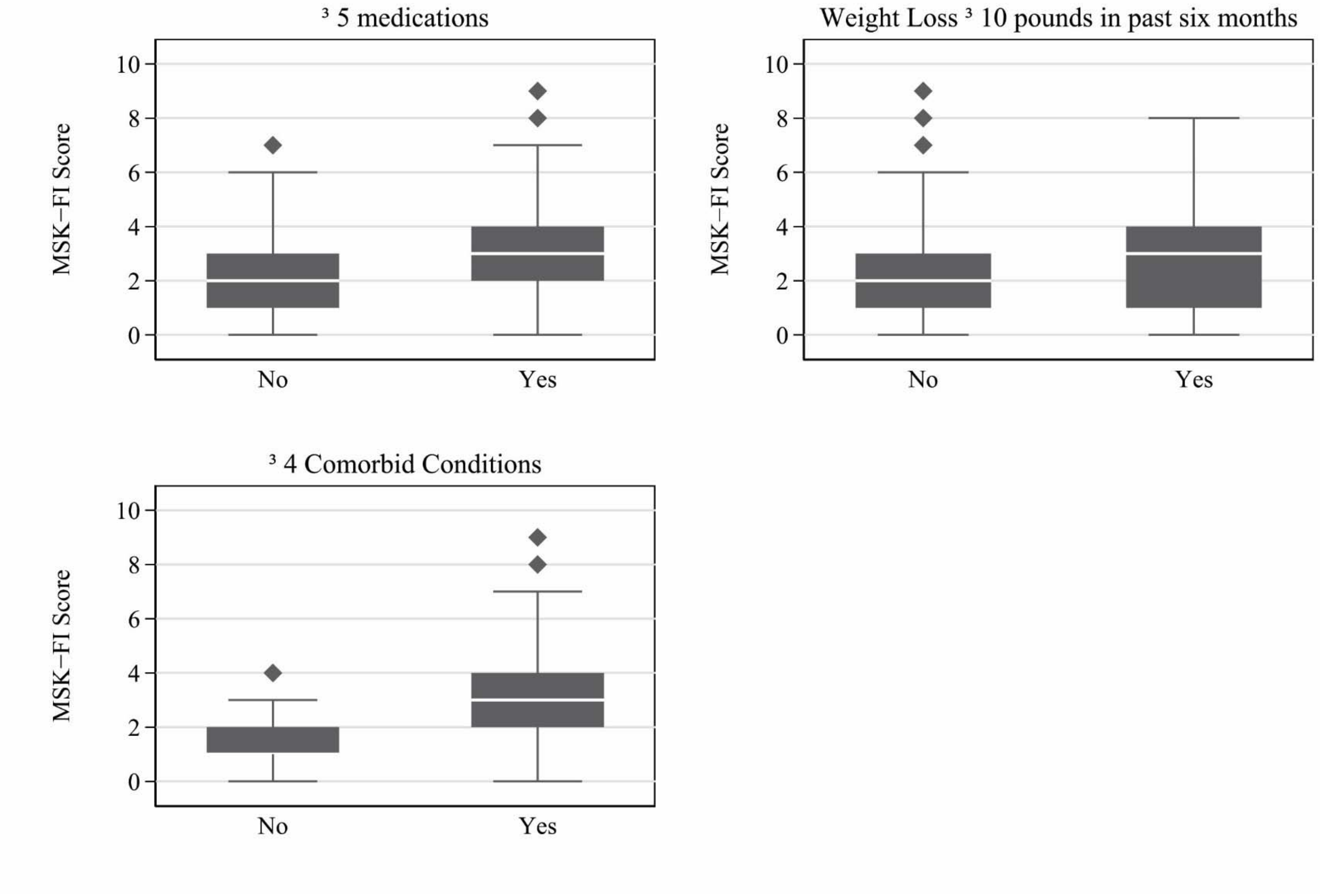

© 2019 Shahrokni A et al. *JAMA Network Open.*

eFigure 4. After Excluding Patients With Stage IV Cancer, Estimated Probability of Death at 12 Months (With Covariates Set at the Mean and Bootstrapped 95% CI)

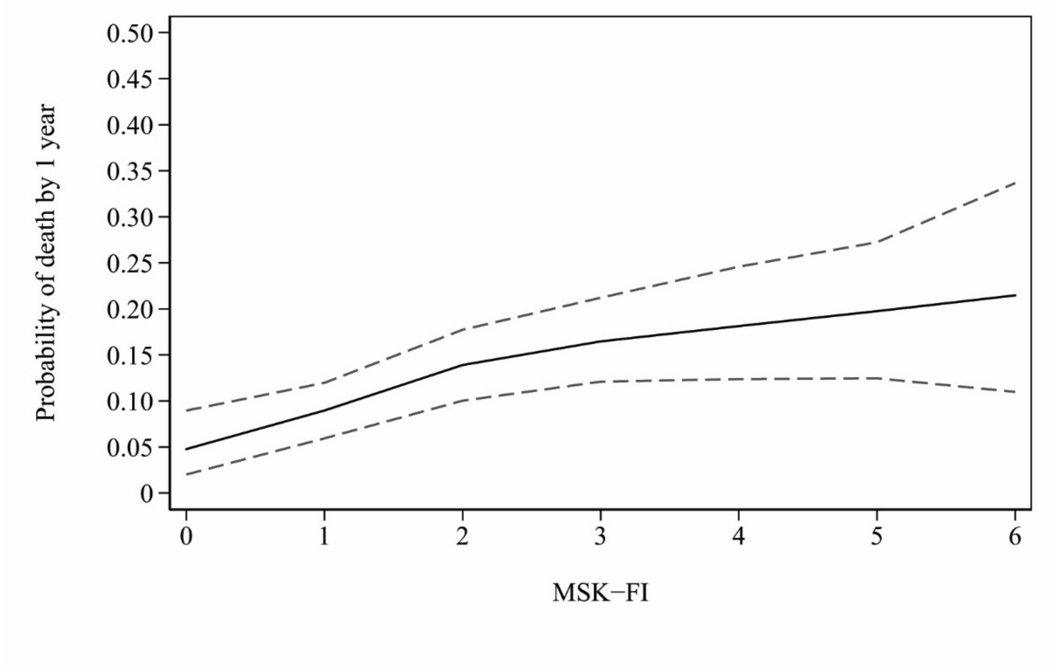

© 2019 Shahrokni A et al. *JAMA Network Open.*

eFigure 5. After Excluding Patients With Stage III or IV Cancer, Estimated Probability of Death at 12 Months (With Covariates Set at the Mean and Bootstrapped 95% CI)

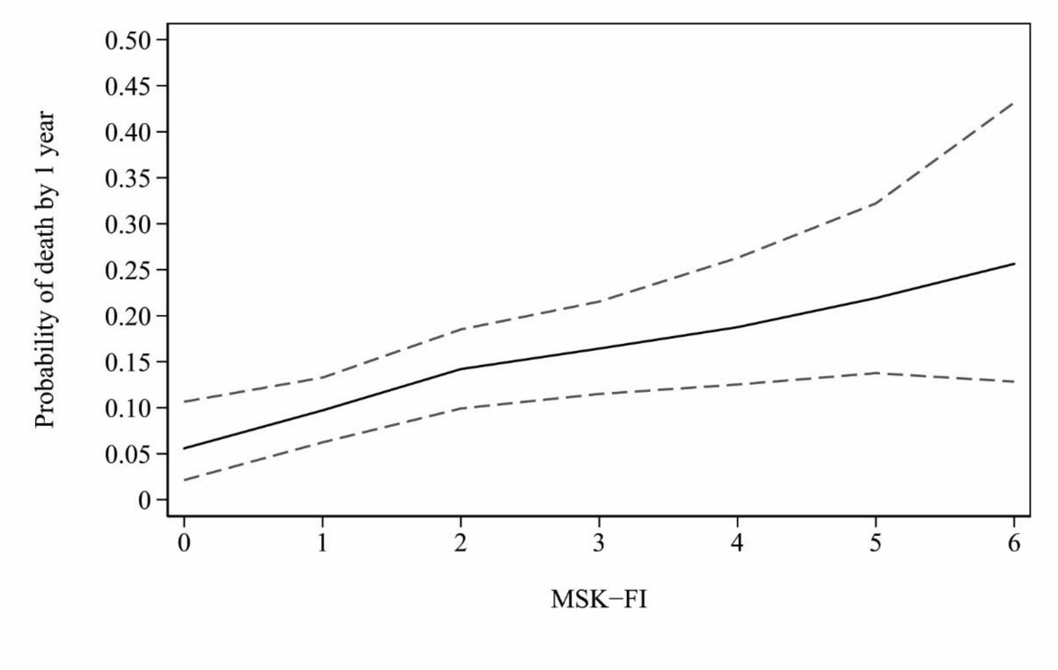

© 2019 Shahrokni A et al. *JAMA Network Open.*

eReferences

1. Katz S. Assessing self-maintenance: activities of daily living, mobility, and instrumental activities of daily living. *J Am Geriatr Soc.* 1983;31(12):721-727.
2. Lawton M, Brody E. Instrumental Activities of Daily Living Scale (IADL). In:1988.
3. Schag CC, Heinrich RL, Ganz P. Karnofsky performance status revisited: reliability, validity, and guidelines. *J Clin Oncol.* 1984;2(3):187-193.
4. Podsiadlo D, Richardson S. The timed “Up & Go”: a test of basic functional mobility for frail elderly persons. *J Am Geriatr Soc.* 1991;39(2):142-148.
5. Gjesfjeld CD, Greeno CG, Kim KH. A Confirmatory Factor Analysis of an Abbreviated Social Support Instrument-The MOSS-SSS. *Research on Social Work Practice.* 2007.
6. Stewart AL. *Measuring functioning and well-being: the medical outcomes study approach.* Duke University Press; 1992.
7. Borson S, Scanlan J, Brush M, Vitaliano P, Dokmak A. The Mini-Cog: a cognitive‘vital signs’ measure for dementia screening in multi-lingual elderly. *Int J Geriatr Psychiatry.* 2000;15(11):1021-1027.
8. Jacobsen PB, Donovan KA, Trask PC, et al. Screening for psychologic distress in ambulatory cancer patients. *Cancer.* 2005;103(7):1494-1502.
9. Almeida OP, Almeida SA. Short versions of the geriatric depression scale: a study of their validity for the diagnosis of a major depressive episode according to ICD-10 and DSM-IV. *Int J Geriatr Psychiatry.* 1999;14(10):858-865.

© 2019 Shahrokni A et al. *JAMA Network Open.*
